# Transfection of brain capillary endothelial cells in primary culture with defined blood–brain barrier properties

**DOI:** 10.1186/s12987-015-0015-9

**Published:** 2015-08-07

**Authors:** Annette Burkhart, Louiza Bohn Thomsen, Maj Schneider Thomsen, Jacek Lichota, Csilla Fazakas, István Krizbai, Torben Moos

**Affiliations:** Department of Health Science and Technology, Laboratory of Neurobiology, Biomedicine Group, Aalborg University, Frederik Bajers Vej 3B, 1.216, 9220 Aalborg East, Denmark; Institute of Biophysics, Biological Research Centre of the Hungarian Academy of Sciences, Szeged, Hungary

**Keywords:** Astrocyte, Blood–brain barrier, Endothelium, Gene therapy, In vitro, Pericytes, Primary culture, Transfection

## Abstract

**Background:**

Primary brain capillary endothelial cells (BCECs) are a promising tool to study the blood–brain barrier (BBB) in vitro, as they maintain many important characteristics of the BBB in vivo, especially when co-cultured with pericytes and/or astrocytes. A novel strategy for drug delivery to the brain is to transform BCECs into protein factories by genetic modifications leading to secretion of otherwise BBB impermeable proteins into the central nervous system. However, a huge challenge underlying this strategy is to enable transfection of non-mitotic BCECs, taking a non-viral approach. We therefore aimed to study transfection in primary, non-mitotic BCECs cultured with defined BBB properties without disrupting the cells’ integrity.

**Methods:**

Primary cultures of BCECs, pericytes and astrocytes were generated from rat brains and used in three different in vitro BBB experimental arrangements, which were characterised based on a their expression of tight junction proteins and other BBB specific proteins, high trans-endothelial electrical resistance (TEER), and low passive permeability to radiolabeled mannitol. Recombinant gene expression and protein synthesis were examined in primary BCECs. The BCECs were transfected using a commercially available transfection agent Turbofect™ to express the red fluorescent protein HcRed1-C1. The BCECs were transfected at different time points to monitor transfection in relation to mitotic or non-mitotic cells, as indicated by fluorescence-activated cell sorting analysis after 5-and 6-carboxylfluorescein diacetate succinidyl ester incorporation.

**Results:**

The cell cultures exhibited important BBB characteristics judged from their expression of BBB specific proteins, high TEER values, and low passive permeability. Among the three in vitro BBB models, co-culturing with BCECs and astrocytes was well suited for the transfection studies. Transfection was independent of cell division and with equal efficacy between the mitotic and non-mitotic BCECs. Importantly, transfection of BCECs exhibiting BBB characteristics did not alter the integrity of the BCECs cell layer.

**Conclusions:**

The data clearly indicate that non-viral gene therapy of BCECs is possible in primary culture conditions with an intact BBB.

## Background

The blood-brain barrier (BBB) denotes the interface between the circulation and the central nervous system (CNS). It is formed by non-fenestrated brain capillary endothelial cells (BCECs) that control the flux of substances into the CNS. Other non-neuronal cells of the CNS, mainly astrocytes and pericytes, form the neurovascular unit together with BCECs and support the function of the BBB to restrict both paracellular and transcellular transport pathways to the CNS [1–4]. The restriction for paracellular transport is mediated via the robust expression of tight and adherence junction proteins between BCECs. These junctions lead to the formation of major gradients for inorganic and organic solutes between the brain and the blood plasma. This accounts for the existence of high transcellular endothelial electric resistance (TEER) across BCECs [5]. Transcellular transport across BCECs is diminutive with respect to hydrophilic molecules [1–4]. Furthermore, the expression of several efflux transporter proteins like the ATP-binding cassette transporters prevents exogenous substances that are lipophilic in nature to pass through the BCECs [6–8].

In order to enable high-throughput screening for permeability of putative CNS acting drugs, much effort has been devoted to designing reliable in vitro models of the BBB. Primary BCECs have been isolated from a variety of animal sources [2, 3, 9–15], which has also resulted in establishment of immortalised cells lines. These have been widely used to study the BBB, but unfortunately, they seem to lose many important BBB characteristics in culture [16–18]. The biggest challenge is to maintain the in vivo characteristics of BCECs with respect to high TEER and low passive permeability when cultured in vitro, and primary isolated BCECs are the most promising tool to study the BBB in vitro [4, 16–19]. An additional way to mimic the microenvironment is by culturing BCECs together with astrocytes and pericytes as co- or triple cell cultures. These culturing conditions significantly up-regulate the expression of important tight junction proteins like zona occludens 1 (ZO1) and claudin-5, which lead to intended higher TEER values and low passive permeability [2, 3, 15, 19].

Gene therapy to BCECs, choroid plexus epithelium and ependymal have been proposed as a promising drug delivery strategy for the delivery of otherwise non-permeable proteins into the CNS [20–22]. The strategy involves genetically modifying the BCECs to express and secrete a protein of interest into the CNS [23, 24]. Previous studies using this strategy have primarily studied gene therapy in mitotic immortalized BCECs without any seemingly BBB properties, using a gene carrier of non-viral origin [23–26]. Non-viral gene carriers are generally believed to be highly dependent on cell division for delivery of genetic material to the nucleus [27–29]. However, in vivo the BCECs are non-mitotic [30], which denotes a major hazard for gene transfection.

A remaining challenge in the future perspectives of genetically modifying BCECs into protein factories is therefore to transfect non-mitotic primary BCECs with defined BBB properties. It would be necessary to investigate whether non-mitotic BCECs are receptive for delivery of genetic material by a non-viral carrier, and subsequently process the encoded protein, without compromising the barrier properties of the BCECs. This was therefore, the main aim of the present study using a plasmid encoding the red fluorescent protein HcRed1-C1.

An in vitro BBB model using primary cells isolated from rat brains was established. Three different models were established and characterised with respect to TEER values, permeability and expression of cell-specific markers. A co-culture model of BCECs and astrocytes was the most stable model and was subsequently used to determine that non-mitotic BCECs with defined barrier properties were receptive for delivery of genetic material by a non-viral carrier, and subsequently processed the encoded protein, without compromising barrier properties of the BCECs.

## Methods

### Materials

Poly(vinylpurrolidone)-iodine complex (Cat. No. PVP1), Percoll (Cat. No. P1644), collagen type IV (Cat. No. C5533), fibronectin (Cat. No. F1141), poly-l-lysine (Cat. No. P6282), heparin (Cat. No. H3149), puromycin (Cat. No. P8833), hydrocortisone (Cat. No. H4001), CTP-cAMP (Cat. No. C3912), 4-(3-Butoxy-4-methoxybenzyl)imidazolidin-2-one (RO-201724) (Cat. No. B8279), competent CG5 Escherichia coli (Cat. No. G3169), 4′, 6-Diamidino-2-phenylindole dihydrochloride (DAPI) (Cat. No. D9542) and mouse anti-alpha-smooth muscle actin (α-SMA) (Cat. No. A5228) were obtained from Sigma-Aldrich (Brondby, Denmark, DK). DNase I (Cat. No. 10104159001), Collagenase/Dispase (Cat. No. 109113), insulin transferrin sodium selenite (Cat. No. 11074547001) and basic fibroblast growth factor (bFGF) (Cat. No. 1363697) were from Roche (Hvidovre, Denmark, DK). Rabbit anti-ZO1 (Cat. No. 61-7300), Alexa Fluor 488-conjugated goat anti-rabbit IgG (Cat. No. A11034), Alexa Fluor 594-conjugated goat anti-mouse IgG (Cat. No. A11032), collagenase II (Cat. No. 17101105), Dulbecco’s Modified Eagle Medium consisting of nutrient Mixture F-12 (DMEM/F-12) (Cat. No. 31331), DMEM (Cat. No. 21885) and fetal calf serum (Cat. No. 10270) were from Life Technology (Naerum, Denmark, DK). Bovine serum albumin (BSA) (Cat. No. EQBAH62) was from Europa Bioproducts (Cambridge, United Kingdom, UK). Plasma derived bovine serum (Cat. No. 60-00-810) was from First Link (Wolverhampton, United Kingdom, UK). Gentamicin sulphate (Cat. No. 17-518Z) was from Lonza Copenhagen (Vallensbaek Strand, Denmark, DK). Fluorescence mounting media (Cat. No S3023) and rabbit anti-glial fibrillary acidic protein (GFAP) (Cat. No. Z0334) were from DAKO (Glostrup, Denmark, DK). ^3^H D-Mannitol (Cat. No NET101250UC) and Ultima Gold™ liquid scintillation cocktail (Cat. No. 6013326) were from Pelkin Elmer (Skovlunde, Denmark, DK). Turbofect™ (Cat. No. R0531), and reagents for qPCR were from Thermo Scientific, except primers synthesised by TAG Copenhagen (Frederiksberg, Denmark, DK). Clonetech pHcRed1-C1 plasmid (Cat. No 632415) and Promokine Cell proliferation kit I (CFDA SE) were from BioNordika Denmark A/S (Herlev, Denmark, DK). Macherey–Nagel NucleoBond^®^Xtra Midi EF plasmid DNA purification kit (Cat. No. 740410) was from AH diagnostics (Aarhus, Denmark, DK). Hanging cell culture inserts (Cat. No. Pirp 15R48) were from Merck Milipore (Hellerup, Denmark, DK).

### Cell culture

Primary cultures of BCECs were prepared from 2 to 3 weeks old Sprague–Dawley rats using slight modifications of the protocol of Nakagawa et al. [19]. The procedures dealing with the handling of rats as described in this study were approved by the Danish National Council of Animal Welfare. One isolation required nine to twelve rats, which resulted in approximately 5–8 million endothelial cells. The rats were anesthetised by subcutaneous injection of 0.5 ml/10 g body weight of Hypnorm/Dormicum (Fentanyl/Fluanisone mixed with Midazolam and sterile water in ratio of 1:1:2). Heads were rinsed with 70% ethanol and 10% poly(vinylpurrolidone)-iodine complex before decapitation. Under sterile conditions, brains were gently dissected, and forebrains collected in ice-cold PBS. Care was taken to remove meninges and any visible white matter, before the cerebral cortices were cut into small pieces using sterile razor blades. The tissue was digested in collagenase II and DNase I in DMEM-F12 at 37°C for 75 min until terminated by diluting in DMEM-F12 followed by centrifugation at 1,000*G* for 8 min. The pellet was resuspended in 20% BSA in DMEM-F12 and centrifuged at 1,000*G* for 20 min. Microvessels present in the pellet were further digested in collagenase/dispase and DNase I in DMEM/F12 at 37°C for 50 min. The digested microvessel fragments were separated on a continuous 33% Percoll gradient. The microvessel fragments were seeded on collagen type IV and fibronectin coated 35 mm plastic dishes. Primary cultures of BCECs were maintained in DMEM/F12 supplemented with 10% plasma derived bovine serum, bFGF, heparin, insulin–transferrin–sodium selenite and gentamicin sulphate and cultured in an incubator with 5% CO_2_/95% air at 37°C. Puromycin was added to the culture media (4 µg/ml) for the first 2 days to obtain a pure culture of BCECs, which in contrast to pericytes are able to thrive due to their high expression of efflux pumps that scavenges the intracellular toxicity generated by puromycin [31].

Primary cultures of pericytes were obtained by prolonged culture of isolated microvessel fragments. These microvessel fragments contain both BCECs and pericytes; however, by culturing the microvessel fragments on uncoated dishes in DMEM supplemented with 10% fetal calf serum and gentamicin sulphate for about 10 days, pericyte survival and proliferation was favoured and BCECs died. The pericytes were frozen in DMEM supplemented with 30% FCS and 7.5% DMSO for later use. They were thawed and cultured for 3 days before being used in the experiments.

Primary cultures of astrocytes were obtained from neonatal Sprague–Dawley rat pups. The pups were rapidly decapitated, their brains dissected and pieces of the cerebral cortex mechanically dissociated through a 40 µm nylon strainer in DMEM supplemented with 10% fetal calf serum and gentamicin sulphate. Dissociated cells were seeded in poly-l-lysine coated culture flasks for approximately 2 weeks until they reached confluence. Thereafter, the cells were either frozen or seeded directly in poly-l-lysine coated 12 well culture plates for about 2 weeks before being used for co-culture experiments with BCECs and pericytes. It was consistently found that the freezing step could be performed without reduction in the cells capacity to influence their inductive effects on the barrier formation of BCECs.

### Construction of in vitro BBB models

Three in vitro BBB models were prepared: monocultures of BCECs, non-contact co-cultures of BCECs and astrocytes, and triple cultures consisting of BCECs, pericytes and astrocytes. Three days after isolation, BCECs reached about 80% confluence and were passaged onto collagen type IV- and fibronectin-coated 12 well polyethylene terephthalate, 1.0 µm hanging cell culture inserts at a cell density of 1 × 10^5^ cells/cm^2^. The cells were left to adhere to the inserts overnight. To construct non-contact cultures, BCECs were seeded on the upper side of the inserts, before the inserts were placed in 12 well culture plates containing a confluent layer of astrocytes. To construct triple cultures, pericytes were seeded on the bottom side of the coated inserts at a cell density of 1.5 × 10^4^ cells/cm^2^ and left to adhere for 4–5 h, before BCECs were seeded on the upper side. The inserts were placed in 12 well culture plates containing confluent layer of astrocytes grown at the bottom of the wells. To further induce BBB characteristics, BCECs were treated with hydrocortisone, cAMP and RO-201724 in concentrations of 550 nM, 250 µM and 17.5 µM respectively [4, 32].

### Immunocytochemistry

Cells were washed in 0.1 M PBS, pH 7.4, fixed with absolute ethanol/acetic acid in a ratio of 95:5% for 10 min at −20°C. They were washed 3 × 5 min in 0.1 M PBS followed by blocking non-specific binding of primary antibodies using 3% BSA in PBS for 30 min at room temperature. BCECs were incubated with antibodies raised against ZO1 (endothelial cells), α-SMA (pericytes), or GFAP (astrocytes). These primary antibodies were used at a dilution of 1:200 in 1% BSA/0.1 M PBS, pH 7.4 and incubated overnight at 4°C. Alexa Fluor 488-conjugated goat anti-rabbit IgG and Alexa Fluor 594-conjugated goat anti-mouse IgG secondary antibodies were used at a dilution of 1:200 in 1% BSA in 0.1 M PBS, pH 7.4 and incubated for 30 min at room temperature. Nuclei were stained with DAPI. The cells were mounted on glass slides with fluorescent mounting medium, and examined in a fluorescence microscope (Axiovert 2000, Carl-Zeiss, Germany). Captured images were corrected for brightness and contrast using Adobe Photoshop C2 software.

### Trans-endothelial electrical resistance (TEER) measurements

TEER was measured to evaluate the integrity of the in vitro BBB models. TEER, which in culture conditions reflects the flux of mainly sodium ions through an intact cell layer, was measured using a Millicell ERS-2 epithelial Volt-Ohm meter and STX01 Chopstick Electrodes (Millipore, Hellerup Denmark, DK). The TEER values of coated but cell-free inserts were subtracted from the measured TEER values, and the difference was multiplied with the size of the insert (1.12 cm^2^). Measured TEER values are given as Ω cm^2^. Data were analysed with GraphPad Prism 5.0 software (GraphPad Software, Inc., CA, USA) using two-way ANOVA with Bonferroni post hoc test.

### Passive permeability across the BCECs

Functional integrity of the BBB models was determined using radiolabeled mannitol [33]. ^3^H-D-Mannitol (specific activity 14.2 Ci/mmol) was added to the upper chamber in concentration of 1 µCi/ml and incubated with cells for 2 h at 37°C on a rocking table. Donor samples (100 µl) were taken from the upper chamber at 0 and 120 min, and receiver samples (100 µl) were taken from the lower chamber at 0, 15, 30, 60 and 120 min and replaced with 100 µl fresh media. Samples were added with Ultima Gold Scintillations fluid and counted in a LKB Wallac Rackbeta Liquid Scintillation Counter, Model 1209. The permeability studies were performed on eighteen inserts on the second day of high TEER (day 2), and the permeability data were plotted as the total number of millimoles transported against time in each well. The flux at steady state across the inserts was calculated as the slope of the straight line at steady state divided by the area of the insert. The apparent permeability (Papp) was calculated by dividing the observed flux at steady state (J) with the initial concentration in the donor compartment (C_donor_). The Papp values were plotted against TEER for the individual inserts.

### RT-qPCR

Gene expression analyses were performed on BCECs cultured in mono-, co- and triple culture conditions. For each RNA sample, RNA was isolated from 20 individual inserts and pooled into four samples each containing five inserts. This procedure was further repeated twice to yield cells from two different isolations, which resulted in eight RNA samples (n = 8) for each of the three different cell culture conditions. RNA was extracted from BCECs using the GeneJET RNA Purification Kit and treated with DNase I enzyme according to the manufacture’s protocol. 100 ng of each DNA-free RNA sample was used as a template for RT-qPCR. cDNA synthesis was carried out with the RevertAid Premium First Strand cDNA Synthesis Kit. To assess the expression profile of BCECs specific proteins, quantitative RT-PCR was performed with primers specific for claudin-5, occludin, platelet/endothelial cell adhesion molecule 1 (PECAM-1), Transferrin receptor 1 (CD-71), ATP-binding cassette, sub-family G, member 2 (ABCG2), also known as breast cancer resistance protein (BCRP) and ATP-binding cassette, sub-family B, member 1 (ABCB1), also known as p-glycoprotein (PgP) (Table 1). Beta-actin was used as housekeeping control gene for normalisation purpose. 2.5 ng cDNA and 10 pmol of each primer were used for each PCR reaction together with the Maxima™ SYBR Green qPCR Master Mix. Each sample was performed in triplicates, and not reverse-transcribed RNA and water served as negative controls. Quantitative RT-PCR was performed using the Stratagene Mx 3000P™ QPCR System (Agilent Technologies, Horsholm, Denmark, DK). The PCR conditions were 95°C for 10 min, followed by 40 cycles of: 95°C for 30 s, 60°C for 30 s and 72°C for 30 s. The relative quantities of DNA in the analysed samples were calculated by the Pfaffl method [34]. Data were analysed by the GraphPad Prism 5.0 software using one-way ANOVA with Tukey’s multiple comparisons post hoc test.

**Table 1:** Primer sequences used for RT-qPCR analysis

Primer	Reference sequence	Forward primer	Reverse primer
*Claudin*-*5*	NM_031701.2	*CTACAGGCTCTTGTGAGGACTTGAC*	*AGTAGGAACTGTTAGCGGCAGTTTG*
*Occludin*	NM_031329.2	*CTGACTATGCGGAAAGAGTCGACAG*	*AGAGGAATCTCCTGGGCTACTTCAG*
*PECAM*-*1*	NM_031591.1	*ATTCTATAAGGACGATGCGCTGGTG*	*GCTGTTCAGTATCACGGTGCATTTG*
*TfR 1*	NM_022712.1	*TGGATCAAGCCAGATCAGCATTCTC*	*TTTCTTCCTCATCTGCAGCCAGTTT*
*ABCG2*	NM_181381.2	*GAGTTAGGCCTGGACAAAGTAGCAG*	*AAGAGGATGGAAGGGTCAGTGATGA*
*ABCB1*	NM_012623.2	*AATCAACAGTACACAGACCGTCAGC*	*CCAAAGTGAAACCTGGATGTAGGCA*
*HcRed1*-*C1*	pHcRed1-C1 (613-1377)	*ATGTACATGGAGGGCACCGTGAA*	*GTCACGTGGATTCTCATGCTCTGG*
*β*-*actin*	NM_031144.3	*CCTCTGAACCCTAAGGCCAACCGTGAA*	*AGTGGTACGACCAGAGGCATACAGGG*

### Measurement of endothelial cell proliferation

BCECs cell division was measured using the PromoKine cell proliferation kit I (CFDA SE), according to manufactures protocol. CFDA SE is also known as CFSE. The fluorescent tracer (495/519) passively diffuses into cells where it binds covalently to intracellular proteins. CFDA SE becomes fluorescent after hydrolysis by intracellular esterases, which results in long term labelling of the cell. This label is inherited through successive cell divisions, and with every cell division, each of the two daughter cells receives approximately half of the label [35]. To analyse the cell division pattern of BCECs, isolated microvessels were seeded directly onto collagen type IV- and fibronectin-coated inserts. The cells were maintained in 4 µg/ml puromycin media for 2 days. One day after isolation (day −2) the cells were incubated with pre-warmed 1 µM CFDA SE in PBS for 15 min to label the cells. Then labelling solution was replaced with pre-warmed medium for 30 min to ensure sufficient hydrolysis of CFDA SE. Labelled cells were cultured for 0–5 days (day −2 to day 3) to allow for cell division. Every 24 h, labelled cells were terminated by detaching the cells of four individual inserts with fixation in 4% paraformaldehyde. At day 0 the cells appeared confluent, growing as a solid monolayer without cells growing on top of each other. They were therefore co-cultured with astrocytes and BBB characteristics were induced by supplementing the media with hydrocortisone, cAMP and RO. TEER was measured daily. Cell divisions were tracked using fluorescence-activated cell sorting (FACS) Canto™ flow cytometer (BD bioscience, Albertslund, Denmark, DK). Prior to the flow cytometic analysis the instrument acquisition parameters were calibrated using FACS 7 colour beads (BD bioscience). The cells were gated using forward and side scatters to eliminate cell debris. Unlabelled BCECs were negative control. The results were analysed using the FlowJo V10 software (TreeStar, Ashland, OR, USA).

### In vitro transfection of BCECs with HcRed1-C1

A plasmid encoding the protein HcRed1-C1 [23] was propagated into competent CG5 E-coli strain by heat shock and purified with ion exchange chromatography using the NucleoBond^®^Xtra Midi EF plasmid DNA purification kit according to the manufacture’s protocol. A commercially available transfection reagent Turbofect™ was used for transfection. For the transfection studies, we took a novel culturing approach and seeded the isolated microvessels directly on collagen type IV- and fibronectin-coated inserts to ease their handling at the time point when they were transferred to wells with astrocytes grown in the lower chamber. The endothelial cell preparation was maintained in 4 µg/ml puromycin media for 2 days. Transfection was performed at different time points after isolation to obtain two different experimental conditions: non-confluent cells (T_−1_), and confluent cells with defined barrier properties (TEER >130 Ω cm^2^) [36] (T_1_). Cells added with transfectants at time point T_1_ were then transferred to grow in co-culture with astrocytes and further supplemented with hydrocortisone, cAMP and RO to induce barrier properties. Cells grown at T_−1_ culture conditions were cultured without astrocytes, hydrocortisone, cAMP and RO.

For each insert, 1 μg plasmid was mixed with 100 μl medium without serum and 2 μl Turbofect™. The solution was incubated at room temperature for 15–20 min for complexes to form. The Turbofect™ solution (102 μl) was added to the luminal compartment in droplets that were dispersed throughout the wells, and the cells were cultured for 48 h in an incubator at 37°C with 5% CO_2_/95% air. TEER was measured daily to access the effects of transfection on the integrity of the cultures. Non-transfected cells served as control for TEER values. TEER data was analysed in the GraphPad Prism 5.0 software using 2-way ANOVA with Bonferroni post hoc test.

After transfection cells were fixed in 4% paraformaldehyde for 10 min at room temperature and stained with DAPI. Some inserts were, additionally, stained for ZO1, as described above. The inserts were removed from their plastic supports with razor blades and mounted on glass slides using fluorescent mounting medium. The HcRed1-C1 protein encoded by the HcRed1-C1 plasmid excites fluorescence excitation and emission maxima at 588 nm and 618 nm.

The transfection efficiency was analysed using RT-qPCR with primers specific for HcRed1-C1 (Table 1). Primers specific for claudin-5 were used to assess the origin of the transfected cells. Beta-actin was used as normalisation control. Non-transfected cells, not-reverse transcribed RNA and water served as negative controls. For each RNA sample, RNA was collected from four individual inserts. This was repeated twice to obtain cells from two different isolations; which resulted in sample values of six or eight RNA samples for all conditions [T_−1_, T_1_ and non-transfected cells (T_CRTL_)]. Data were analysed by the GraphPad Prism 5.0 software using a 1-way ANOVA with Tukey’s multiple comparisons post hoc test.

The transfection efficiency was further analysed using Flow cytometry. At the end of transfection, cells were washed in PBS, detached from the insert and fixed in 4% paraformaldehyde. Cells expressing red fluorescence protein HcRed1-C1 were counted using the MoFlo^®^ Astrios™ Flow cytometer system (Beckman Coulter, Copenhagen, Denmark, DK). Prior to the flow cytometric analysis the instrument parameters were calibrated using SPHERO™ ultra rainbow fluorescent particles (3 μm) (Spherotech, Lake Forrest, IL, USA). Cells were gated using forward and side scatters to eliminate cell debris. HcRed1-C1 positive cells were gated based on auto fluorescence from unlabelled BCECs to ensure less than 1% false positive events occurred. The results were analysed using FlowJo V10 software.

## Results

### Establishment of BBB in primary culture

**Fig. 1:**
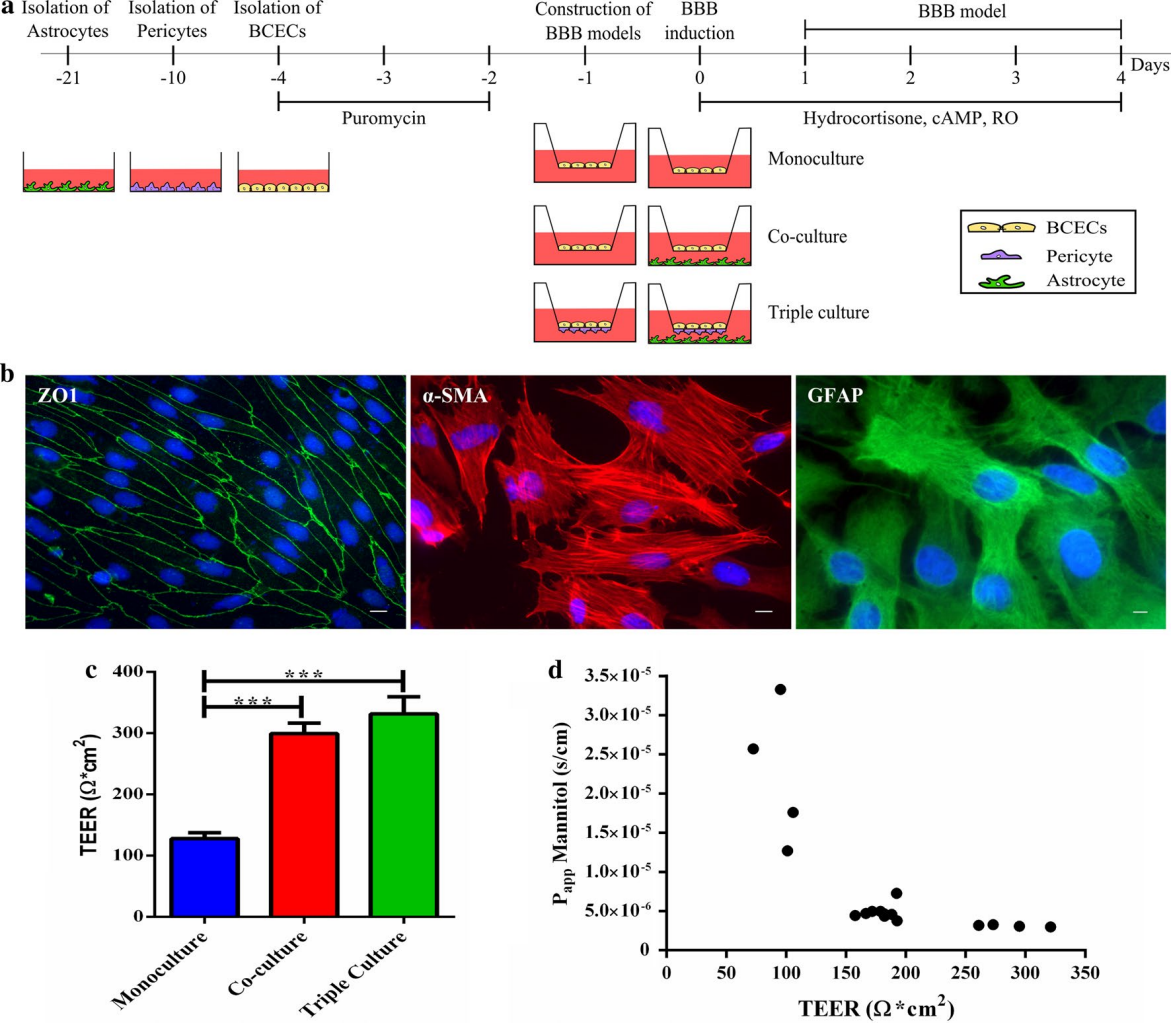
Establishment and characterization of in vitro BBB models. **a** Monoculture consisting of brain capillary endothelial cells (BCECs, *yellow*), a non-contact co-culture of BCECs and astrocytes (*green*) and a triple culture of BCECs, pericytes (*purple*) and astrocytes. Astrocytes and pericytes were cultured for 21 and 10 days, respectively. The BCECs were cultured for 3 days until 80% confluent. Puromycin was added to the media for the first 2 days. On day −1 the pericytes and/or endothelial cells were passaged to each side of the inserts and left to adhere for 24 h. On day 0, the inserts used for co- and triple culturing were moved to a plate containing astrocytes, and the BCECs were stimulated with hydrocortisone, cAMP and RO. **b** The cells were identified based on their expression of the tight junction protein ZO1 (BCECs), alpha smooth muscle actin (α-SMA) (pericytes) and glial fibrillary acidic protein (GFAP) (astrocytes). The cell nuclei were counterstained with DAPI (*blue*). *Scale bars* 10 µm. **c** The maximal TEER values were reached on day 2. Co-cultures (*red*), and triple cultures (*green*) displayed TEER values of 299 ± 17 and 331 ± 28 Ω cm^2^ respectively, while the monoculture (*blue*) only showed a slight increase in TEER (128 ± 9 Ω cm^2^). Data are presented as means ± SEM (n = 24). Statistical differences were analysed using a 1-way ANOVA with Tukey’s multiple comparisons post hoc test (***p < 0.001). There was no ignificant difference between co- and triple cultures. **d** The apparent permeability (Papp) of mannitol in cultured BCECs. Data are calculated based on measurements from 18 inserts with TEER values ranging from 72.4 to 321 Ω cm^2^. The permeability to mannitol decreases as TEER values increase around 150 Ω cm^2^, which can be obtained by co-culturing the BCECs with pericytes and/or astrocytes.

Three cell types were used to construct the three different types of in vitro BBB models (Fig. 1a). Based on their respective expression of the cell specific markers ZO1, α-SMA and GFAP, the three isolated cell types were identified as BCECs, pericytes and astrocytes, respectively (Fig. 1b). Evaluating the integrity of the BCECs in these in vitro models, the TEER measurements increased in all cultures in consequence to stimulation with hydrocortisone, cAMP and RO-201724. On day 2, the mono-, co- and triple culture models reached their maximal TEER values of 128 ± 9, 299 ± 17 and 331 ± 28 Ω cm^2^, respectively (Fig. 1c). TEER values of co- and triple cultures were significantly higher than in monocultures, indicating the importance of co-culturing the BCECs with astrocytes and pericytes. The TEER values of triple culture were generally higher than that of the co-cultures albeit this difference was not statistically significant. A large variability was seen in the TEER values obtained with the triple culture (SEM ± 28) compared to the mono- (SEM ± 9) and co-culture (SEM ± 17) models. Both co- and triple cultures maintained TEER above 150 Ω cm^2^ until day 4, while monocultures failed to even reach 130 Ω cm^2^ at any experimental day. TEER measurements were not conducted beyond day 4, since TEER values decreased below 130 Ω cm^2^, at which stage in vitro BBB models using rat endothelial cells get insufficiently tight [36]. Evaluating the permeability of the endothelial monolayer to mannitol demonstrated that TEER values around 150 Ω cm^2^ and upwards clearly resulted in lower permeability to mannitol, which was did not decrease further, with higher TEER values. This is therefore in agreement with the observations reported by Gaillard and de Boer [36], and indicates that TEER values around 130–150 Ω cm^2^ is sufficient to obtain a low permeability (Fig. 1d).

**Fig. 2:**
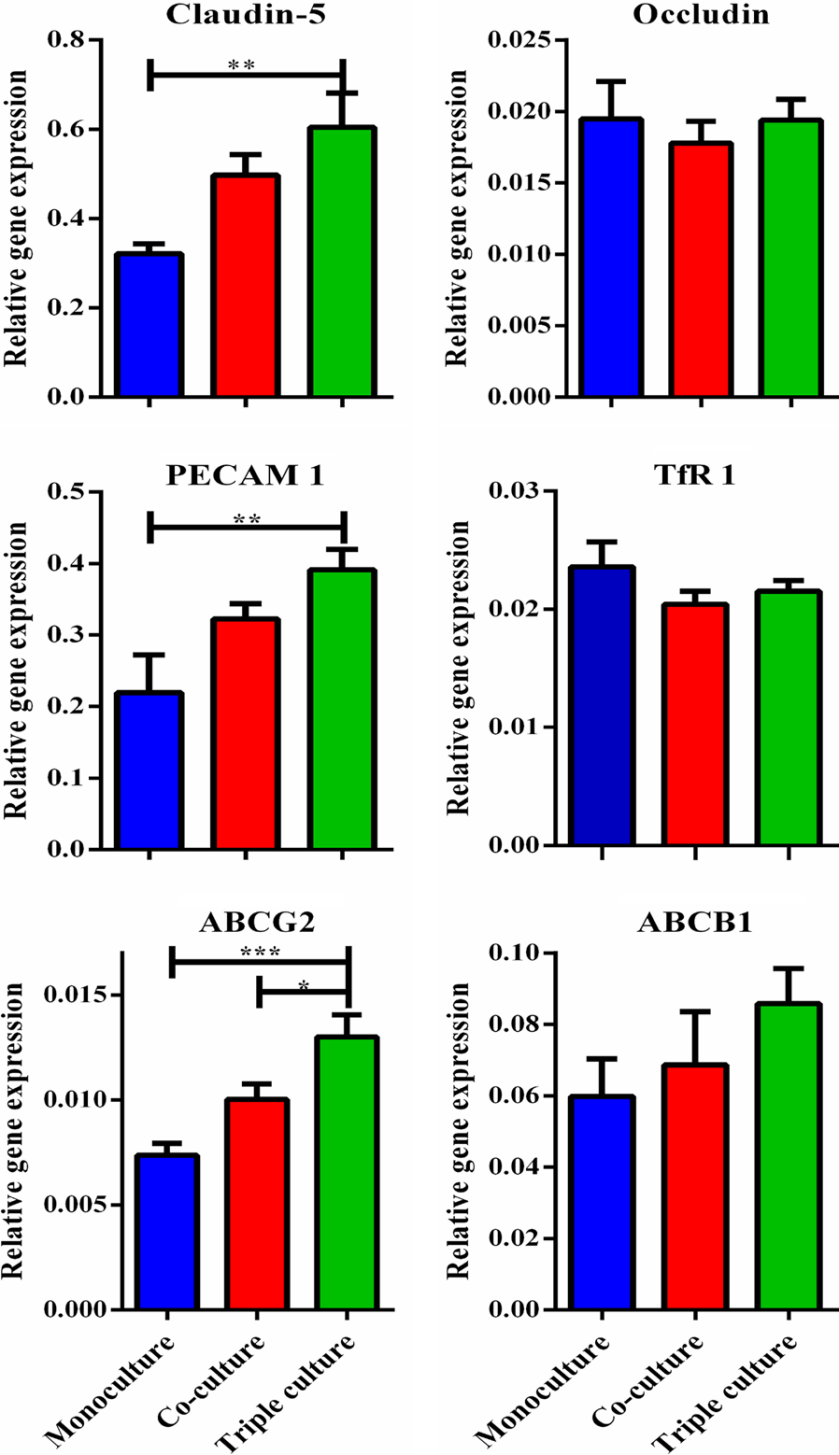
Gene expression analysis of the hall mark proteins related to brain capillary endothelial cells (BCECs). RNA was obtained from BCECs grown in mono- (*blue*), co- (*red*) and triple- (*green*) culture conditions at day 1 and analysed for the expression of BCECs hallmark genes (claudin-5, occludin, PECAM-1, ABCG2, ABCB1 and Transferrin receptor 1 (TfR1)). The relative gene expression among the three culture conditions was statistically analysed using 1-way ANOVA with Tukey’s multiple comparisons post hoc test. Data are presented as sample means ± SEM (n = 8). p < 0.05, **p < 0.01, ***p < 0.001.

The in vitro models were also examined for expression of genes signifying BCECs. TEER was measured prior to RNA extraction and was 73 ± 14 Ω cm^2^ for monoculture, 229 ± 34 Ω cm^2^ for co-culture and 243 ± 26 Ω cm^2^ for triple culture. BCECs grown in triple culture had a significantly higher expression of claudin-5 and PECAM-1 compared to the monoculture. Furthermore, ABCG2 was statistically higher expressed in the triple cultures compared to both mono- and co- cultures. This expression correlates well with increasing TEER in the different culture models (Fig. 2). By contrast occludin, transferrin receptor 1 and ABCB1 did not show any statistic difference between groups.

### Cell division among BCECs related to the stage of BBB maturity

**Fig. 3:**
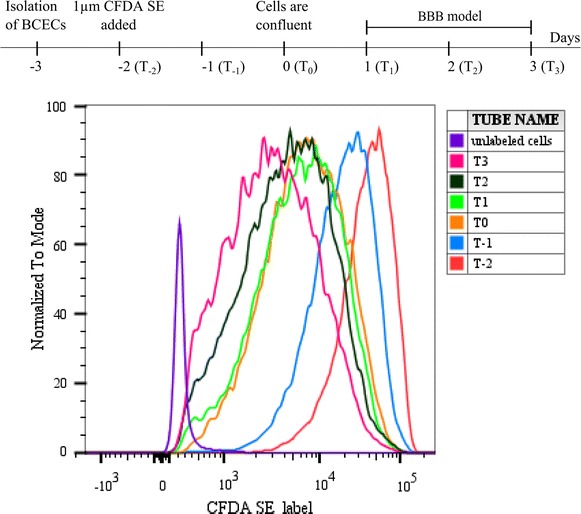
Analysis of the cell proliferation of brain capillary endothelial cells (BCECs) from barrier culture days −2 to 3 using a 5-and 6-carboxylfluorescein diacetate succinidyl ester (CFDA SE) assay. BCECs were isolated on day −3 and seeded directly onto inserts. 1 µM CFDA SE was added to the cells at day −2. After 30 min of incubation all the cells were labelled with CFDA SE and the first group were terminated (T_−2_) (*red*). Every day for 5 days one group (T_−1_ to T_3_) (*blue*,* orange*,* green*,* black* and* purple*, respectively) were terminated. The BCECs were microscopically visualised to be confluent on day 0, and subsequently co-cultured with astrocytes and stimulated with hydrocortisone, cAMP and RO-201724. The cells were examined on BD FACS canto™ and analysed with the FlowJo v10 software. The cells were gated using forward and side scatter to eliminate cell debris. Unlabelled BCECs (*purple*) were used as a negative control.

Since no significant difference was found in TEER between co- and triple cultures, the co-culture was decided sufficient for further analyses concerning cell division and transfection studies. In order to establish a model in which different stages of barrier maturity could be analysed, the setup was slightly modified, and for this the co-culture model was most suitable. The BCECs were seeded directly onto the inserts on the day of isolation (−3) (Fig. 3), visualised by phase contrast microscopy, and confluent by day 0. From day 0 the previous described setup (Fig. 1a) was followed by BBB induction and co-culturing with astrocytes at day 0. BBB properties were present from day 1 to 3 with TEER values well above 130 Ω cm^2^.

The different stages of barrier maturity were defined according to cell confluence and it was, therefore, important to investigate whether BCECs continued their division despite reaching confluence. Newly isolated BCECs were left to adhere to the insert for 24 h by which 1 µM CFDA SE was added to the BCECs on day −2. Every day from day −2 to day 3 cells were terminated and the amount of CFDA SE label was analysed by FACS. The intensity of CFDA SE was largely reduced from day −2 until day −1 and again from day −1 to day 0, indicating that BCECs were highly dividing (Fig. 3). After day 0 the proliferation seemed to dramatically decrease as the BCECs retained approximately the same degree of CFDA SE label. This observation corresponded well to the microscopic observations of BCECs reaching confluence at day 0. The curves corresponding to day 1 to day 3 widened, indicating a more diverse population of BCEC division judged from their different CFDA SE label. There was a small reduction in the CFDA SE label on day 3 representing a small degree of cell division at this stage. The change in the level of CFDA SE label from day 1 to day 3 was, however, far from that seen from day −2 to day 0.

### Transfection of BCECs in primary culture

**Fig. 4:**
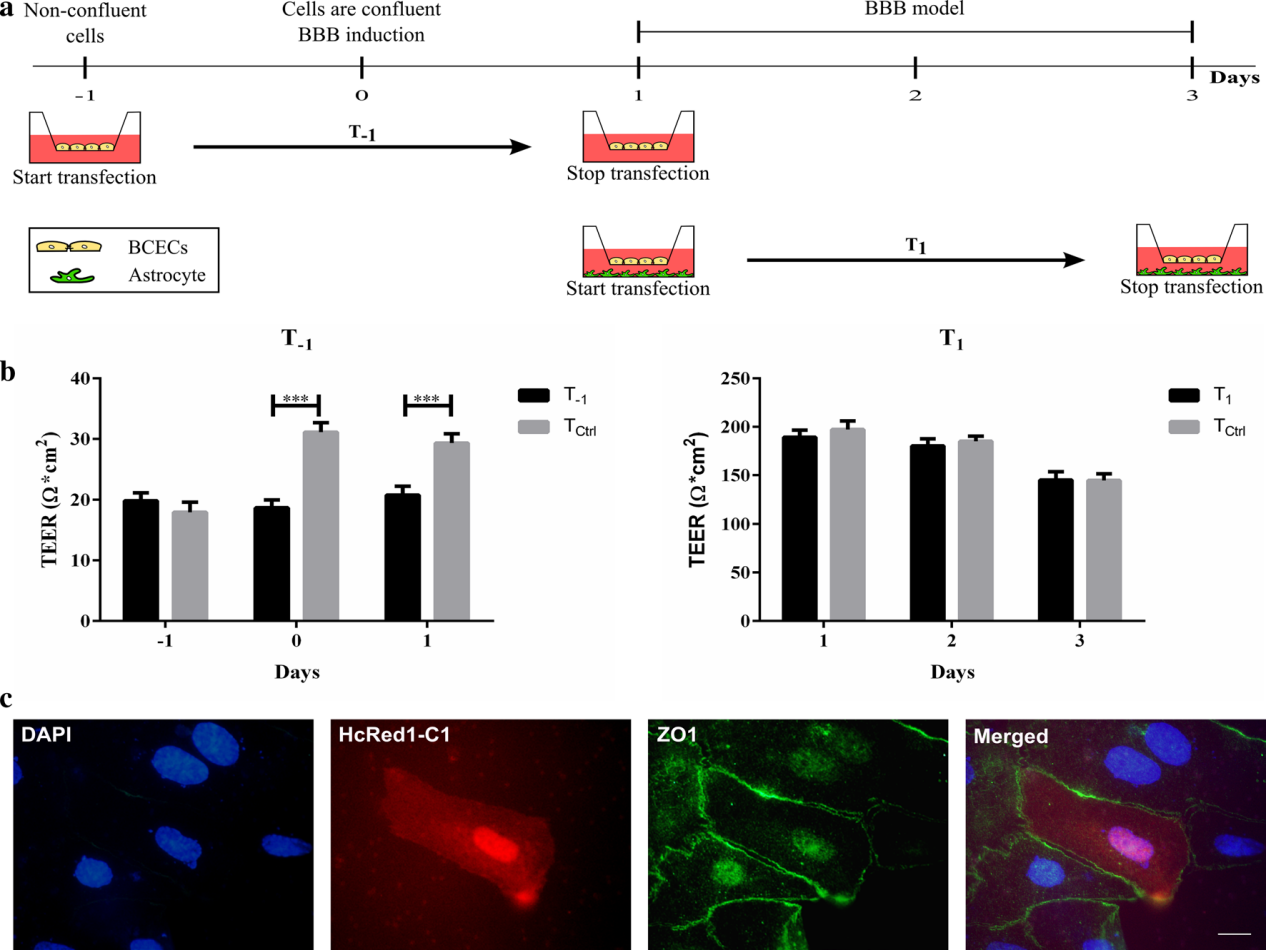
The effect of transfection on the integrity of the brain capillary endothelial cells (BCECs). **a** The experimental design used for in vitro transfection of BCECs. BCECs (*yellow*) were isolated on day -3, seeded directly onto the inserts. Barrier properties were induced by co-culturing with astrocytes (*green*) in the presence of hydrocortisone, cAMP and RO-201724. BCECs were transfected at two different stages of barrier maturity: T_−1_, an immature state, defined by dividing BCECs without barrier properties, and T_1_, a mature state defined as the BCECs being confluent and having barrier properties. **b** The integrity of the transfected BCECs (T_−1_ and T_1_) (*black*) was monitored daily by measurements of TEER and compared to non-transfected cells (T_CTRL_) (*grey*). *Left* non-confluent BCECs transfected on day −1 (T_−1_). No attempts were made to increase tight junction formation. *Right* on experimental day 1, barrier properties were present (TEER above 150 Ω cm^2^) in both transfected and control BCECs and lasted for at least 2 days. Significant differences among the two states and their respective controls were analysed using 1-way ANOVA with Tukey’s multiple comparisons post hoc test (***p < 0.001). No significant difference was found between T_1_ and T_CTRL_. Data are presented as means ± SEM (n = 34–44). **c** To investigate the origin of the HcRed1-C1 positive cells, an immunocytochemical analysis was performed for the tight junction protein ZO1. The cells illustrated were transfected at day 1 (T_1_) and examined 48 h after transfection. The illustrations depict a BCEC containing both the HcRed1-C1 protein and the ZO1 protein (*green*). Nuclei are counterstained with DAPI (*blue*). *Scale bar* 10 µm.

The model established by culturing the BCECs on the inserts was found to exhibit clear signs of BBB integrity for approximately 2 days after initiating the co-culture between BCECs and astrocytes. Therefore, a 48 h time window was available to conduct the transfection experiments. The freshly isolated primary BCECs were seeded directly onto inserts and two different stages of BBB maturity were defined according to the modified setup (Fig. 3). The two stages were: Stage T_−1_, which is defined as the immature state with the BCECs still undergoing cell division, and T_1_, the mature state where the BCECs grown in co-culture with astrocytes had established true barrier properties. Only stage T_1_ was co-cultured with astrocytes, as transfection at the immature state (T_−1_) was initiated prior to the day of BBB induction (Fig. 4a). The BCECs were transfected with a plasmid encoding the red fluorescence HcRed1-C1 protein and TEER was measured daily. Significantly lower TEER values were found at the immature stage (T_−1_) compared to non-transfected cells (T_CTRL_) in the same condition (Fig. 4b, left). No significant differences in TEER were found between BCECs transfected at the mature state (T_1_) compared to correspondingly grown non-transfected cells (Fig. 4b, right). Both of the BCECs preparations had TEER values ranging above the critical 130 Ω cm^2^ at the beginning of transfection needed to form a tight barrier in the rat (T_1_: 189 ± 7 Ω cm^2^, T_CTRL_ 197 ± 9 Ω cm^2^), and these TEER values stayed above 130 Ω cm^2^ until the end of the observation period denoted by 48 h. after initiating the transfection (T_1_: 145 ± 9 Ω cm^2^, T_CTRL_ 145 ± 7 Ω cm^2^) (Fig. 4b, right).

**Fig. 5:**
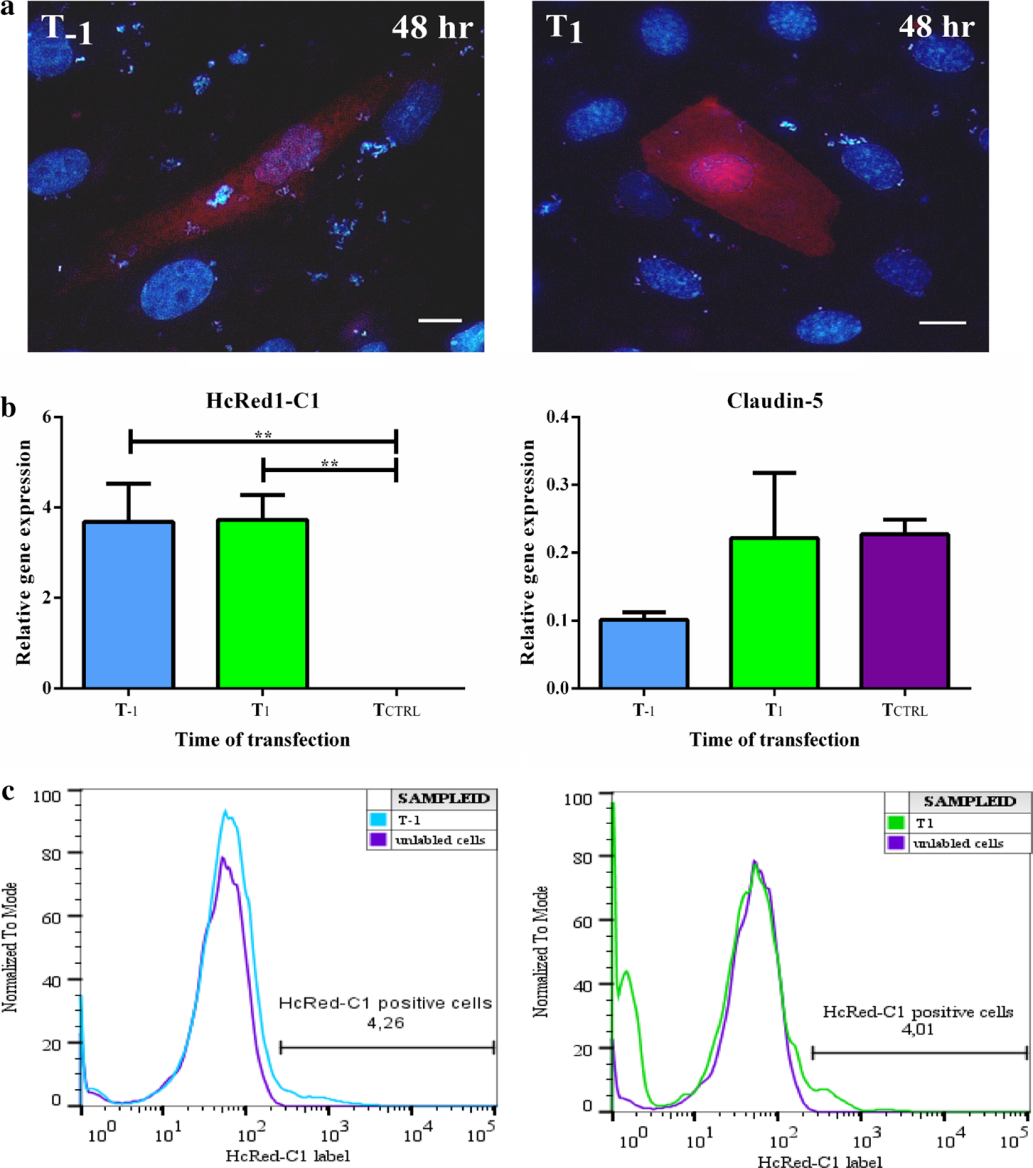
The transfection efficiency of brain capillary endothelial cells (BCECs) at different stages of barrier maturity. **a** Expression of HcRed1-C1 fluorescent protein as seen in BCECs 48 h after transfection. HcRed1-C1 positive cells were expressed at both stages of barrier maturity. The HcRed1-C1 protein distributed to both the cell nucleus and cytoplasm. *Scale bar* 10 µm. **b** The relative gene expression of HcRed1-C1 was investigated with RT-qPCR at the two different states of barrier maturity: T_−1_ (*blue*) and T_1_ (*green*). Non-transfected cells (T_CTRL_) (*purple*) showed no HcRed1-C1 gene expression. The gene expression of claudin-5 was included to evaluate the origin of the transfected cells and to measure the degree of tight junction formation at the various stages of barrier maturity. The relative gene expression among the three culture stages was statistically analysed using 1-way ANOVA with Tukey’s multiple comparisons post hoc test (**p < 0.01). Data are presented as sample means ± SEM (n = 6–8). No significant differences were found in the expression pattern of claudin-5 among the three groups. **c** The transfection efficacy of BCECs was additionally assessed by flow cytometry. A transfection efficacy of about 4% was found in both the immature highly diving stage (T_−1_) and in the mature non-dividing stage (T_1_). Results were analysed using the FlowJo V10 software. The cells were gated using forward and side scatter to eliminate cell debris. Additionally, the cells were corrected for auto fluorescence using unlabelled BCECs (purple) to ensure that less than 1% of the HcRed1-C1 positive cells were false positive. *Scale bar* 10 µm.

HcRed1-C1 protein distributed to both cytoplasm and the nucleus of BCECs (Figs. 4c, 5a), which indicated that the HcRed1-C1 encoding plasmid was successfully delivered to the cell nucleus and expressed by the BCECs independently of barrier maturity. Pericyte contamination of BCECs could not be excluded, although the amount of pericytes was determined to be very low. Immunolabeling of transfected cells was performed after transfection and this labelling showed that the HcRed1-C1 positive cells also expressed the tight junction protein ZO1, which identifies them as BCECs (Fig. 4c). The cells depicted were transfected at day 1 (T_1_); however, ZO1 and HcRed1-C1 positive cells were found in both transfection setups (data not shown).

### Correlation between barrier properties and transfection efficiency

The HcRed1-C1 gene expression by BCECs was evaluated by RT-qPCR analysis and confirmed gene expression in the two culture conditions (Fig. 5b). The expression after T_−1_ and T_1_ transfection was 3.7 ± 0.8 and 3.7 ± 0.6, respectively. HcRed1-C1 gene expression was not found in non-transfected cells (T_CTRL_). The expression of the claudin-5 gene was, additionally, included in this analysis to simultaneously evaluate the endothelial origin of the transfected cells and the degree of tight junction formation. Claudin-5 was present in all three situations. No significant differences were found in the expression pattern of claudin-5 among the three groups, although there was a tendency towards claudin-5 being lowest in the immature state (T_−1_), which correlated with the lower TEER value.

The transfection efficacy was furthermore analysed by flow cytometry (Fig. 5c). The BCECs were transfected with the red fluorescent HcRed1-C1 protein at the two different stages of barrier maturity for 48 h. Unlabelled cells were used to assess the auto fluorescence and to ensure that less than 1% of the HcRed1-C1 positive cells were false positive. A transfection efficiency of approximately 4% was found both in the immature dividing state (T_−1_) and in the mature state (T_1_).

## Discussion

### Establishment of the in vitro BBB model

BCECs were cultured in monolayer, and the effects of co-culturing the BCECs with astrocytes and pericytes were analysed based on TEER values, and gene expression profiles using a variety of BBB specific proteins. The passive permeability was analysed in relation to TEER and clearly demonstrated that TEER values around 150 Ω cm^2^ were sufficient to obtain low permeability, as the co-cultures reached TEER values near 300 Ω cm^2^ that coincided with low passive permeability. This corresponds very well to the data presented by Gaillard and de Boer, where they found that as TEER values above 130 Ω cm^2^, were sufficient to establish a tight in vitro BBB model using primary cells isolated from rat brains [36]. As the TEER values in monocultures of the BCECs never exceeded 130 Ω cm^2^, this culture did not reach the demands for a tight in vitro BBB model. Astrocytes had the highest impact on BCECs as only minor, non-significant effects was seen when culturing BCECs in presence of both astrocytes and pericytes with regard to TEER values.

Astrocyte-endothelial interactions are important for establishment and regulation of BBB properties of BCECs in vitro [1, 5, 37]. This mimicking recapitulated many features of the BBB in vivo; a dramatic increase in TEER due to increased expression of tight junction proteins [15, 19, 32], lowering of passive permeability [19] and up-regulation of important nutrient transporters [38, 39]. The fact that the addition of astrocytes to the BCECs to develop the co-culture raised the TEER value substantially shows that astrocytes play a pivotal role for induction of BBB properties in vitro. Pericytes also induce the BBB phenotype in BCECs cultured in vitro [2, 16, 19], although the data of the present study did not find evidence that pericytes, when added to astrocytes formed significantly improved BBB characteristics of BCECs.

That BCECs did not statistically increase TEER under influence of pericytes could reflect the differential state of the pericytes. The expression of α-SMA by pericytes affect the BBB properties of BCECs, as α-SMA positive pericytes are unable to increase TEER, whereas the reverse applies to α-SMA negative pericytes [40]. When grown in monoculture pericytes differentiate into α-SMA negative state when exposed to bFGF but becomes α-SMA expressing in the presence of transforming growth factor β (TGFβ) [40]. When culturing the pericytes together with BCECs and astrocytes in triple culture, they invariably get exposed to both bFGF and TGFβ secreted by astrocytes [5, 41–43]. Moreover, bFGF was added to the cell media used for BCECs. Possibly, the influence of TGFβ secreted by astrocytes drives pericytes towards their α-SMA expressing state that subsequently leads to less BBB inductive functions. The differentiation state of the pericytes was, therefore, difficult to control and might have resulted in different differentiation of pericytes, resulting in large variability in the TEER values.

TEER values of the co and triple cultures increased until day 2 after which they started to decrease. Hence, TEER values remained high above 130 Ω cm^2^ for at least 48 h. after stimulating the cells to increase their expression of tight junction proteins. A single study reported that TEER values remained high for more than 5 days in non-transfected conditions [2], while another group report that the TEER values of non-transfected BCECs decrease after day 3 [44]. We have a suspicion that differences in maintaining the barriers between different research groups could derive from variations in the handling of the cell cultures, e.g. the use of the chopstick for measuring TEER demands the removal of the cells from the incubator multiple times. This invariably affects the buffer capacity of the media [33], which consequently may loosen the astrocytic cell layer from the culture wells. Supporting our notion, when non-transfected BCECs were co-cultured with astrocytes and left undisturbed in the incubator for 3 days, the endothelial cells maintained their TEER value around 180 Ω cm^2^, which clearly suggests that handling the co-cultures can negatively affect the barrier integrity.

### Transfection of BCECs in primary culture

For transfection purposes, the BCECs were grown directly on filters, which ensured that only BCECs would take up the transfections agent but also that the cells could undoubtedly be characterised as primary cells (P0). However, this led to a lower, albeit still clearly acceptable, high TEER value indicative of good integrative BBB properties. TEER values were generally lower when the BCECs were seeded directly onto the filters, compared to when they were passaged on the filters 3 days after isolation. We believe this could relate to the presence of cell debris and capillary fragments on the filters, which were otherwise removed during the passaging step. However, TEER invariably remained above 130 Ω cm^2^ indicating that the BBB integrity was clearly maintained during the transfection situation. Based on co-culture conditions, we introduced genetic material into BCECs at different states of barrier maturity, which enabled us to monitor correlations between cell division, barrier maturity and transfection efficiency. Cells undergoing mitosis are more prone to gene therapy than non-dividing cells [45]. However, the majority of BCECs are post-mitotic in vivo [30], which complicates the introduction of new genetic material [46]. This problem can be overcome using a viral vector with mechanisms for nuclear internalisation in non-dividing cells [46]. Despite high efficiency for cellular insertion of genetic material including cells of the CNS [47–50]; viral vectors may, however, also exert unwanted effects [51]. We therefore investigated transfection efficiency of BCECs taking a non-viral approach using Turbofect, a cationic polymer that forms stable and positively charged complexes with DNA. According to the manufacturer, Turbofect has low cytotoxicity, is independent of serum-free conditions, and considered suitable for transfection of primary cells.

No difference was found in the percentage of transfected cells, which in both cases were around 4%. Interestingly, BCECs grown in the mature state of the BBB also took up HcRed1-C1 gene material and transcribed it without influencing the tightness of the cell layer. This observation suggests that transfection may be independent of cell division and that transfection of non-dividing cells with barrier properties is as effective as transfection of dividing cells without barrier properties.

Previous transfection studies on BCECs were performed in monocultures without polarised conditions [23, 26, 52, 53]. We performed transfection under polarised conditions by adding genetic material to luminal side. Theoretically, this material could pass though tight junction complexes and enter BCECs via the abluminal side. This option was, however, not likely when considering the size of the plasmid-Turbofect complex and the high expression of tight junction proteins. Supporting this notion, we found no expression by astrocytes present on the abluminal side of BCECs.

Cytoplasmic delivery is one of several obstacles in the design of drug carriers. The ultimate challenge could be the entry of genetic material into the nuclear envelope. Molecules smaller than approximately 40 kDa are able to diffuse passively into the nucleus via nuclear pore complexes, while macromolecules larger than 60 kDa require a nuclear localisation sequence. The size of plasmid DNA makes it unlikely that nuclear entry occurs through passive diffusion [54]. The transfection efficacy has been reported to be higher in mitotic cells than in quiescent counterparts [27, 45, 46], which has led to the idea that plasmid DNA enters the nucleus during disassembly of the nuclear envelope during mitotic cell division [45, 54]. Studies investigating the correlation between mitotic activity and transfection efficacy revealed that transfection was probable even when cells were arrested in the G1phase [27, 45, 46], which indicates that plasmid DNA might permeate the nuclear pore complexes by a mechanism that resembles the active transport of polypeptides larger than 60 kDa [29, 54]. Therefore, transfection of BCECs when they enter their quiescent stage may relate to active transport of plasmid DNA though the nuclear pore complex not directly related to cell division.

Many therapeutic polypeptides are acknowledged for their neuroprotective effects [55, 56]. Prospects of using BCECs as protein factories to enable transport of neuroprotective polypeptides into the brain without a need to pass the BBB are substantial [22]. The present study is the first to demonstrate non-viral gene therapy to BCECs in cultures with defined BBB properties. The present study revealed transfection efficacies around 4%. It is however questionable whether this percentage of transfected cells would be enough to see any therapeutic effects in vivo. In comparison transfection of HeLa cells with identical plasmid and transfection agent results in a transfection efficiency of 46% (unpublished results). We are therefore currently testing other non-viral gene carriers in order to increase the transfection efficiency of the non-mitotic BCECs. Further studies also need to examine the potential of secretion from BCECs transfected with DNA encoding neuroprotective proteins like recombinant erythropoietin and brain derived neurotrophic factor (BDNF). Nonetheless, the current study is the first crucial step in the evaluation of the use of non-viral gene therapy to non-mitotic BCECs with defined BBB properties as a potent future drug delivery strategy to the CNS.

## Conclusions

In conclusion, non-viral transfection of polarised BCECs with defined BBB properties enabled gene expression even in conditions with low mitotic activity, hence simulating the in vivo condition. Transfection did not disrupt the BBB integrity judged from the conserved expression of tight junction proteins. Moreover, transfection was independent of cell division, and as effective as transfection of dividing cells without barrier properties. The data clearly indicate that non-viral gene therapy of BCECs is possible in culture conditions with an intact BBB. The data also suggest that genetically modification of BCECs in vivo might be a feasible drug delivery strategy in the future.

## Abbreviations

α-SMA: alpha-smooth muscle actin
ABCB1: ATP-binding cassette, sub-family B, member 1
ABCG2: ATP-binding cassette, sub-family G, member 2
BBB: blood–brain barrier
BCECs: brain capillary endothelial cells
bFGF: basic fibroblast growth factor
BCRP: breast cancer resistance protein
BSA: Bovine serum albumin
CFDA SE: 5-and 6-carboxylfluorescein diacetate succinidyl ester
CNS: central nervous system
DAPI: 4′,6-diamidino-2-phenylindole dihydrochloride
FACS: fluorescence-activated cell sorting
GFAP: glial fibrillary acidic protein
Papp: apparent permeability
PBS: phosphate buffered saline
PECAM-1: platelet/endothelial cell adhesion molecule 1
PgP: p-glycoprotein
TEER: trans-endothelial electrical resistance
TGFβ: Transforming growth factor β
RO-201724: 4-(3-Butoxy-4-methoxybenzyl)imidazolidin-2-one
ZO1: zona occludens 1
